# Incidence of stress fractures of the femoral shaft in women treated with bisphosphonate

**DOI:** 10.3109/17453670903139914

**Published:** 2009-08-01

**Authors:** Jörg Schilcher, Per Aspenberg

**Affiliations:** Department of Orthopedics, AIM/IKE, Faculty of Health Science, Linköping UniversityLinköpingSweden

## Abstract

**Background** Recent case reports have identified an association between long-term bisphosphonate treatment and stress fractures of the femoral shaft. The risk of such fractures in bisphosphonate users has not been determined.

**Methods** We identified women over 55 years of age with the specific fracture pattern by searching the operation registry of the hospitals in 2 healthcare districts. Prevalence of bisphosphonate treatment was provided by a Swedish national registry covering all drugs delivered to all individuals since 2005.

**Results** The number of women on bisphosphonate treatment was 3,087. Of these, 5 had femoral stress fractures. They had been taking bisphosphonates for 3.5 to 8.5 years. The incidence density for a patient on bisphosphonate was 1/1,000 per year (95% CI: 0.3–2). In the remaining 88,869 women who were not taking bisphosphonates, there were 3 stress fractures. Thus, their risk (without correction for inhomogeneity in age distribution) was 46 times less (95% CI: 11–200).

**Interpretation** These results are rough estimations based on a comparatively small material. Still, a treatment-associated incidence density of 1/1,000 is acceptable, considering that bisphosphonate treatment is likely to reduce the incidence density of any fracture by 15/1,000 according to a large randomized trial (Black et al. 1996).

## Introduction

There are several case reports on transverse fractures of the femoral shaft in patients who are on bisphosphonate treatment (Goh et al. 2007, Kwek et al. 2008a, Kwek et al. 2008b, Lenart et al. 2008, Neviaser et al. 2008, Sayed-Noor and Sjoden 2008). These fractures show cortical thickening as a sign of a stress fracture. They are often preceded by local pain for some weeks before a sudden break with minimal trauma. Many cases are bilateral.

Most of these patients have taken a bisphosphonate for several years (Neviaser et al. 2008). However, there have been no reports on the incidence of these fractures in patients on bisphosphonates, so the size of the risk is unknown.

Our 4 hospitals take care of all fractures within their catchment areas (with 570,000 inhabitants in total, 420,000 of which are in the county of östergötland). The Swedish health authorities have registry data on all drugs taken by every individual in the population. We could therefore calculate the annual incidence of femoral shaft stress fractures in patients taking bisphosphonates.

## Patients and methods

All femoral shaft fractures with the diagnosis codes S72.30 and S72.20 (ICD10 clinical coding system) in women over 55 years of age were collected by searching the operation registry database of the university hospitals in Lund and Linköping. The Linköping database also covers the other 2 hospitals in the county of östergötland. These hospitals take care of all trauma in their catchment areas. The time period evaluated for Lund was from January 2007 to May 2008 and for Linkoping from January 2007 to October 2008. All preoperative radiographs were examined by JS to identify stress fractures. Suspected stress fractures were then confirmed by PA, without prior discussion. This identification was done without any knowledge of drug treatment. The stress fracture pattern, described by Lenart et al. (2008) as “simple with thick cortices”, was defined as a transverse fracture of the femoral shaft with cortical thickening (Figure).

**Figure:**
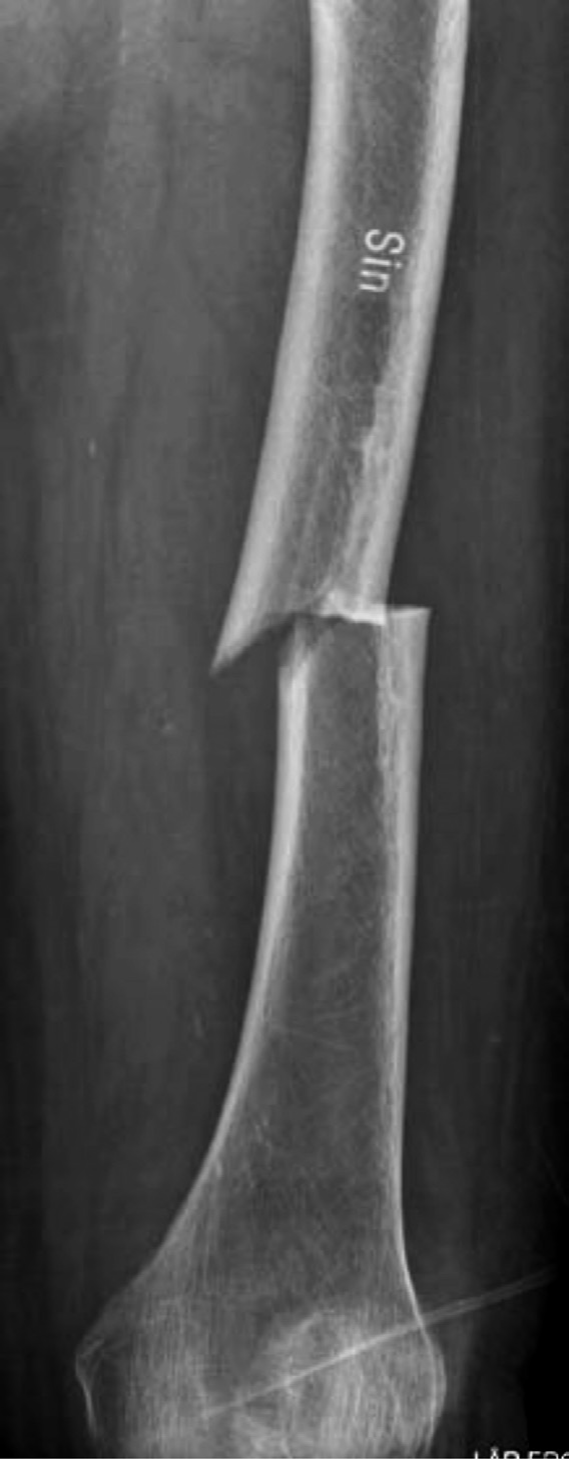
Typical stress fracture in one of the bisphosphonate-treated patients. Note the transverse fracture line on the lateral side and the cortical thickening.

The Swedish National Board of Health and Welfare provided prevalence data for continuous treatment with bisphosphonate drugs with the ATC codes M05BA and M05BB (M05BA: bisphosphonate drugs; M05BB: combinations of bisphosphonate drugs with calcium) in the catchment areas of the hospitals. This database covers all pharmaceutical dispensing to every patient in Sweden, and was introduced in July 2005. Continuous treatment was defined as the patient having purchased the drug at least once every year from the introduction of the registry until July 2008. Statistics Sweden, a central government authority for official statistics, provided population data for the districts of interest in the study. Because both the population size and the number of bisphosphonate prescriptions were relatively constant over the years, we have regarded the data as reasonable estimations for incidence densities.

The medication status of each patient with a femoral stress fracture was evaluated by direct telephone contact with the patient. When data were unclear, we contacted the health service responsible and obtained treatment data from electronic patient records.

## Results

We identified 8 patients with stress fractures of the femoral shaft. 5 of them were on continous treatment with bisphosphonate drugs. All of these patients were over 75 years of age. Mean treatment duration was 5.8 (3.5–8.5) years. One patient, who also had rheumatoid arthritis, had bilateral fractures.

Of the 3 patients with stress fractures but no bisphosphonate treatment, 2 of them probably had poor bone health. One of them suffered from low-energy bilateral femoral shaft fractures 6 years apart. She also suffered a perioperative pertrochanteric fracture while being operated for her first shaft fracture, and had refractures twice at the end of an osteosynthesis plate. Another patient suffered from chronic active autoimmune hepatitis, which was treated with low-dose steroids and mercaptopurine before the stress fracture. In the third untreated patient, no predisposing factors were found. In contrast to the bisphosphonate-treated patients, those without bisphosphonates were all less than 75 years old.

In 2007, the overall population of women older than 55 years in the area of interest was 91,956. Of these, 3,087 (3.4%) were on continous treatment with bisphosphonate drugs. In order to combine the catchment areas of östergötland county and Lund University Hospital and the different time intervals for evaluation, we calculated the annual incidence of femoral shaft stress fractures to be 2.4 fractures per year, 1.5 of which were associated with bisphosphonate treatment. The overall incidence of stress fractures in patients on continuous bisphosphonate treatment (weighted for the different catchment areas) was 1/1,000 per year (95% CI: 0.3–2) as compared to 0.02/1,000 (95% CI: 0.004–0.1) for the untreated women.

Because of the small numbers, it was not possible to calculate the relative risk for separate age groups, although there appeared to be different age distributions for fractures in bisphosphonate users and those who were not on bisphosphonates. As a rough estimate, we therefore calculated the relative risk without age correction, and found a 46-times increased risk of stress fracture with bisphosphonates (95% CI: 11–200).

## Discussion

Is the risk of bisphosphonate-associated fractures so great that treatment should be stopped? Our data suggest that it is not. The annual number of hip fractures in women over 65 in Sweden is roughly 11,000, and the total number of osteoporosis fractures is much higher. Because the total number of women over 65 is about 900,000, our data suggest that if this entire population was given bisphosphonates, this would cause 900 stress fractures. This is clearly less than the number of osteoporosis fractures that would be avoided (Black et al. 1996).

Our calculations have several sources of uncertainty. The reason that we present our results in spite of these uncertainties is that there have been no previous incidence calculations available at all. We calculated the relative risk without correction for inhomogeneity in age distribution.

In the future, calculations should be based on larger populations, and should include such corrections. Our present results should be regarded as a first attempt to define the problem.

All patients with a bisphosphonate-associated stress fracture in our material were over 75 years of age. This may reflect the increasing number of bisphosphonate prescriptions with age, but the numbers are too small for certain conclusions to be reached. On the other hand, Kwek et al. (2007) reported a mean age of 66 years for their 17 patients.

It has been suggested that bisphosphonate treatment should be terminated after 5 years (Black et al. 2006). We could only determine the number of patients within the catchment area who had bisphosphonate treatment for a period of 2–3 years. The number of patients with 5 years of treatment may have been less. In that case, the true incidence density—with 5 years of treatment—might be higher. Not all patients who regularly take out their prescriptions do take their medicine, although the fact that they actually bought their medicine suggests a reasonable degree of compliance. Thus, the stress fracture incidence after 5 years of correct treatment was probably higher than our finding of 1/1,000.

Nevasier et al. (2008) looked at all low-energy femoral shaft fractures at their hospital and found that among bisphosphonate users, the stress fractures occurred after a substantially longer treatment than other types of fractures, indicating that the risk of stress fracture increases with treatment time. Only 1 of their 18 stress fracture patients had taken a bisphosphonate for less than 4 years, and the average duration of treatment was 7 years. We found an average duration of 6 years, and Kwek et al. (2008) 5 years. Taken together, these findings suggest that treatment should be stopped after 5 years. It is not certain that prolongation of treatment for more than 5 years reduces the risk of osteoporosis fracture any further (Black et al. 2006).

We had 2 patients with bilateral fractures, 1 of whom was not on bisphosphonates. Half of Kwek's patients had bilateral fractures (Kwek et al. 2008a). The high incidence of bilaterality suggests that the patients with bisphosphonate-associated stress fractures are derived from a subpopulation of all bisphosphonate-treated patients: if all patients on bisphosphonate in our study had the same incidence density of stress fracture (1/1,000)—and if it were independent of individual factors—the incidence density of 2 fractures would be squared, which would make a bilateral fracture extremely unlikely. The bilateral fractures therefore corroborate the suspicion that patients with bisphosphonate-associated stress fractures carry some other risk factor in addition to taking the drug.
